# Deletion of glutamate carboxypeptidase II (GCPII), but not GCPIII, provided long‐term benefits in mice with traumatic brain injury

**DOI:** 10.1111/cns.14299

**Published:** 2023-06-22

**Authors:** Tongjie Ji, Ying Pang, Meng Cheng, Rui Wang, Xu Chen, Chunyu Zhang, Min Liu, Jing Zhang, Chunlong Zhong

**Affiliations:** ^1^ Department of Neurosurgery Shanghai East Hospital, School of Medicine, Tongji University Shanghai China; ^2^ Institute for Advanced Study Tongji University Shanghai China

**Keywords:** bovine serum albumin, glutamate carboxypeptidase II (GCPII), glutamate carboxypeptidase III (GCPIII), glutamate excitotoxicity, learning and memory, microglia, N‐acetylaspartylglutamate (NAAG), traumatic brain injury

## Abstract

**Main Problem:**

N‐acetylaspartylglutamate (NAAG) has neuroprotective effects in traumatic brain injury (TBI) by activating metabotropic glutamate receptor 3 (mGluR3) and reducing glutamate release. Glutamate carboxypeptidase II (GCPII) is the primary enzyme responsible for the hydrolysis of NAAG. It remains unclear whether glutamate carboxypeptidase III (GCPIII), a homolog of GCPII, can partially compensate for GCPII's function.

**Methods:**

GCPII^−/−^, GCPIII^−/−^, and GCPII/III^−/−^ mice were generated using CRISPR/Cas9 technology. Mice brain injury model was established through moderate controlled cortical impact (CCI). The relationship between GCPII and GCPIII was explored by analyzing injury response signals in the hippocampus and cortex of mice with different genotypes at the acute (1 day) and subacute (7 day) phase after TBI.

**Results:**

In this study, we found that deletion of GCPII reduced glutamate production, excitotoxicity, and neuronal damage and improved cognitive function, but GCPIII deletion had no significant neuroprotective effect. Additionally, there was no significant difference in the neuroprotective effect between the combination of GCPII and GCPIII deletion and GCPII deletion alone.

**Conclusion:**

These results suggest that GCPII inhibition may be a therapeutic option for TBI, and that GCPIII may not act as a complementary enzyme to GCPII in this context.

## Graphical abstract

WT and GCPIII^−/−^ mice exhibited pronounced neuronal glutamate excitotoxicity in the hippocampus during the acute phase (1 day) following TBI, extensive microglia activation during the subacute phase (7 days), and noticeable impairment in learning and memory at 14 days after TBI. Conversely, GCPII^−/−^ and GCPII/III^−/−^ mice displayed significantly milder neurological deficits in comparison. Notably, no significant variations were observed between the WT and GCPIII^−/−^ groups, or between the GCPII^−/−^ and GCPII/III^−/−^ groups, suggesting that GCPII inhibition may be a therapeutic option for TBI, and that GCPIII may not act as a complementary enzyme to GCPII in this context.

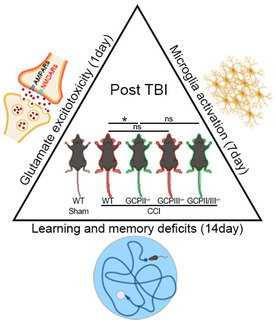

## 1 INTRODUCTION

Glutamate is a vital excitatory neurotransmitter in the central nervous system (CNS) that plays a critical role in cognitive function, memory, movement, and sensation. Excessive glutamate exposure in the CNS can trigger neuronal injury or death, known as glutamate excitotoxicity, which occurs in acute insults and neurodegenerative diseases.^1^, ^2^ Therefore, understanding the mechanisms involved in glutamate signaling regulation that contribute to excitotoxicity is crucial for developing future therapeutic approaches for related diseases.

N‐acetylaspartylglutamate (NAAG) is the third most prevalent neurotransmitter in the mammalian nervous system,^3^ acting as a neuromodulator of glutamatergic synapses by activating presynaptic metabotropic glutamate receptor 3 (mGluR3) and limiting N‐methyl‐D‐aspartate (NMDA) receptor subsets under certain conditions.^4^, ^5^ This activation inhibits the release of glutamate, making NAAG a potential protective agent in traumatic brain injury (TBI).^5^, ^6^ However, the short half‐life of NAAG limits its clinical application, as it is rapidly hydrolyzed into N‐acetylaspartate (NAA) and glutamate by NAAG peptidases in the brain.^7^, ^8^

Glutamate carboxypeptidase II (GCPII), also known as folate hydrolase (Folh1), is a zinc‐dependent acidic amino acid exopeptidase that hydrolyzes NAAG to glutamate and NAA.^9^ Inhibiting GCPII can alter NAAG and glutamate neurotransmission and has been confirmed as a potential therapeutic approach in many animal brain disorder models.^5^, ^6^ We previously demonstrated that treatment with ZJ‐43, a novel GCPII inhibitor, produced significant protection in rats after brain injury by reducing neuron and astrocyte damage 24 h after injury.^10^ We confirmed that ZJ‐43 elevated extracellular NAAG levels and reduced extracellular levels of amino acid neurotransmitters following TBI.^11^ Besides, GCPII knockout (GCPII^−/−^) mice have been reported to develop normally and be less susceptible to oxidative stress, neural damage, and cognitive impairment in the context of experimental ischemic brain injury and TBI.^9^, ^12^, ^13^, ^14^

Glutamate carboxypeptidase III (GCPIII), a homolog of GCPII, was found in the brains, spinal cords, and kidneys of GCPII^−/−^ mice in recent years.^15^ GCPIII has almost the same 3D structure and very similar enzyme activity to GCPII, making it a potential therapeutic target for the treatment of brain disorders as the complementary enzyme to GCPII.^16^, ^17^ Studies have shown that compared with GCPII, GCPIII has lower NAAG hydrolyzing activity and was observed to cleave the physiological substrate β‐citrylglutamate (BCG) with high efficiency.^17^ These results suggest that GCPIII may not merely act as a complementary enzyme to GCPII.

Despite extensive research on the physiological function and pathology of GCPII, there is a lack of experimental data on the role of GCPIII in CNS disorders, such as brain injury. To address this gap, we conducted a study to identify the expression pattern of glutamate carboxypeptidase following injury and investigate the functional effect of GCPII and GCPIII deletion after TBI. With the aid of CRISPR/Cas9‐mediated genome engineering, we generated GCPII^−/−^, GCPIII^−/−^, and GCPII/III^−/−^ mice successfully to evaluate the impact of glutamate carboxypeptidase depletion on major neural cells in the hippocampus during the acute and subacute phase of TBI. Moreover, we attempted to observe the differences in gene expression patterns in the hippocampus of different genotype mice after TBI.

## 2 MATERIALS AND METHODS

### 2.1 Animal

The study utilized male C57BL/6 mice aged 8–12 weeks, purchased from Shanghai SLAC Laboratory Animal Co., Ltd. Additionally, constitutive GCPII knockout (GCPII^−/−^) mice and GCPIII knockout (GCPIII^−/−^) mice were generated by Shanghai Model Organisms Center, Inc., using CRISPR/Cas9‐mediated gene editing on mice with a C57BL/6 background, where exon 2 was targeted for GCPII knockout and exons 3–5 were targeted for GCPIII knockout (Figure 2A). GCPII/III knockout mice (GCPII/III^−/−^) were obtained by crossing GCPII^−/−^ and GCPIII^−/−^ mice. The mice were housed in a suitable environment with a 12‐h light/dark cycle and provided with free access to food and water until the experiment was conducted. Approval for all animal experiments was obtained from the Animal Care and Use Committee of Tongji University School of Medicine.

### 2.2 Controlled cortical impact (CCI)

A mouse model of TBI was established based on previous methods with some modifications.^9^, ^18^ The mice were anesthetized with intraperitoneal injection of sodium pentobarbital (50 mg/kg) and secured in a stereotaxic frame (Stoelting). A sagittal scalp incision was made, followed by a 4 mm diameter craniectomy on the right side of the skull between bregma and lambda, with the middle edge located 2 mm from the midline. Prior to injury, a 3.0‐mm circular impact tip was attached to an electromagnetically controlled impact device (PinPoint™ PCI3000 Precision Cortical Impactor™, Hatteras Instruments) and adjusted to a 15° angle with the vertical plane to ensure perpendicular contact with the dura. In this study, a single impact with the following moderate injury parameters was delivered: piston velocity of 3.0 m/s, deformation depth of 1.0 mm, and impact duration of 180 ms. After the injury was administered, the scalp was sutured, and the mice were placed on a heated blanket for recovery while monitoring body temperature with a rectal thermometer to maintain it at 37°C. Sham mice underwent the same procedures except the impact.

### 2.3 RT–qPCR and Real‐Time RT–qPCR

Total RNA was isolated from the mouse cortex and hippocampus using TRIzol (Invitrogen, USA) according to the manufacturer's protocol and transcribed into cDNA using the PrimeScript™ RT reagent Kit (Takara, RR037A). For RT‐qPCR, amplification was performed using 2 × AceTaq Master Mix (Vazyme, P412‐01), and the resulting products were electrophoresed on a 2% agarose gel. Real‐time RT‐PCR detection was carried out using TB Green® Premix Ex Taq™ II (Takara, RR820A) on the QuantStudio 7 Flex Real‐Time PCR System (Thermo). The relative expression of the transcripts was calculated using the comparative threshold cycle method (ΔΔCt) with Gapdh as the endogenous reference gene. The primer sequences are provided in Table 1.

**TABLE 1:** List of primer pairs used for RT–qPCR and real‐time RT–qPCR.

Primer	Forward (5′–3′)	Reverse (5′–3′)
GCPII	P1‐AGCACCATGAGGTTTGGTTCA	P2‐TCCCAGGTTCACCCTTATCAC
GCPII	P3‐GAATTGAAGGCTGAGAACAT	P4‐ACGTTCCTCAGTCCAAATCT
GCPIII	P1‐TGTTTCTCTCTCCCTCTCCC	P2‐TGTCACTCTTGGCTCTCTCATA
GCPIII	P3‐TTTGGTTGTTCTCCCTGGGT	P4‐CAATGAAAAGGTCTACCTGG
GFAP	CGGAGACGCATCACCTCTG	AGGGAGTGGAGGAGTCATTCG
IBA1	ATCAACAAGCAATTCCTCGATGA	CAGCATTCGCTTCAAGGACATA
Glutaminase	GACAACGTCAGATGGTGTCAT	TGCTTGTGTCAACAAAACAATGT
NR2A	ACGTGACAGAACGCGAACTT	TCAGTGCGGTTCATCAATAACG
NR2B	GCCATGAACGAGACTGACCC	GCTTCCTGGTCCGTGTCATC
TGF‐β1	CCACCTGCAAGACCATCGAC	CCCCTTTCATTCCGCGCTTAT
Gapdh	GGTGAAGGTCGGTGTGAACG	CTCGCTCCTGGAAGATGGTG

### 2.4 Western blot (WB) analysis

The tissue samples were homogenized and solubilized in RIPA lysis buffer (Beyotime), supplemented with 1 mM phenylmethanesulfonylfluoride fluoride (PMSF; Beyotime). Protein samples were separated by electrophoresis and transferred to nitrocellulose membranes (Millipore, Merck KGaA). The membranes were then blocked with 5% Bovine Serum Albumin (BSA) for 1 h at room temperature and incubated overnight at 4°C with primary antibodies. After washing with Tris‐buffered saline with Tween 20 (TBST) three times for 5 min each time, the membranes were incubated with Horseradish Peroxidase (HRP)‐conjugated secondary antibodies for 1 h at room temperature. Related antibodies are shown in Table 2. The signal was detected using Enhanced Chemiluminescence (ECL) substrate (Millipore). Band density was quantified using ImageJ software.

**TABLE 2:** Primary and secondary antibodies used in this study.

Antibody	Lot #	Source	Dilution/Concentration
Mouse anti‐GCPII	GTX80151	GeneTex	1:1000 (WB), 1:200 (IF)
Rabbit anti‐GFAP	80,788	Cell Signal	1:1000 (WB), 1:500 (IHC)
Chicken anti‐GFAP	ab4674	Abcam	1:200 (IF)
Rabbit anti‐IBA1	ab178846	Abcam	1:1000 (WB), 1:200 (IF), 1:500 (IHC)
Rabbit anti‐Caspase3	9662	Cell Signal	1:1000 (WB)
Rabbit anti‐cleaved Caspase3	9664	Cell Signal	1:1000 (WB)
Rabbit anti‐Bax	2774	Cell Signal	1:1000 (WB), 1:200 (IF)
Rabbit anti‐GAPDH	AF7021	Affinity	1:2000 (WB)
Rabbit anti‐beta Actin	AF7018	Affinity	1:2000 (WB)
Goat Anti‐Rabbit IgG HRP	S0001	Affinity	1:5000 (WB)
Goat Anti‐Mouse IgG HRP	S0002	Affinity	1:5000 (WB)
Goat Anti‐Mouse AlexaFluor 488	115–545‐003	Jackson	1:200 (IF)
Goat Anti‐Rabbit AlexaFluor 594	111–585‐003	Jackson	1:200 (IF)
Rabbit Anti‐Chicken AlexaFluor 647	303–605‐003	Jackson	1:200 (IF)

### 2.5 Immunofluorescence staining

The mouse brains were treated with 4% paraformaldehyde (PFA) and then sliced into 30‐μm sections using a vibrating microtome (Leica VT1200S). Three mice were included in each group, and two coronal sections of the middle hippocampal region were randomly selected from each mouse for staining. The number of cells in the hippocampal CA3 region was analyzed statistically. The brain slices were washed with PBS and blocked with permeabilization buffer (0.3% Triton X‐100 in PBS) containing 10% donkey serum for 60 min at room temperature. Primary antibodies were added to the slices and left overnight at 4°C. Then, fluorescent secondary antibodies and 4',6‐diamidino‐2‐phenylindole (DAPI) were added, and the sections were incubated for 1 h at room temperature. Table 2 shows the related antibodies. The sections were then covered with mountain medium (Dako) after being washed. Confocal images were obtained using a confocal microscope (Leica SP8) and processed using SP8 software.

### 2.6 Immumohistochemical staining

The brain tissues were fixed in 4% PFA for 3 h and embedded in wax and then were sectioned at a thickness of 5 μm. The section selection and statistical analysis were conducted in accordance with immunofluorescence standards. The slices were then deparaffinized and hydrated using gradient concentrations of alcohol and xylene. Next, the sections were treated with hot antigen repair solution at 96 to 98°C for 15 min. Endogenous enzyme activity was inactivated with 3% hydrogen peroxide, and nonspecific sites were blocked with goat serum. The tissue was then covered with primary antibody and placed in a wet box at 4°C overnight. The following day, the primary antibody was washed off with PBS, and the corresponding secondary antibody was incubated. Related antibodies are shown in Table 2. After washing the secondary antibody, the sections were stained with 3,3'‐diaminobenzidine (DAB) solution and then washed and dehydrated using gradient concentrations of alcohol and xylene. After drying, the slices were sealed with an appropriate amount of neutral gum and observed under a microscope for analysis.

### 2.7 Analysis of the glutamate concentration

The mice in all groups were euthanized by cervical dislocation 24 h after controlled cortical impact (CCI), and the hippocampus was rapidly harvested, microdissected, frozen in liquid nitrogen, and homogenized using a tissue pulverizer cooled with dry ice. To prepare for liquid chromatography coupled with mass spectrometry (LC–MS), 300 μL of ice‐cold methanol containing 0.1% formic acid was added to each tube. The samples were vortexed for 5 min and centrifuged for 15 min at 13000 rpm at 4°C. The supernatant was filtered with a 0.22‐μm filter membrane, and the filtered supernatant was used for analysis. Glutamate concentration was measured using a mass spectrometer (TSQ quantum triple quadrupole mass spectrometer, Thermo) and chromatograph (Ultimate 3000rs, Thermo).

### 2.8 Nissl staining

Nissl staining was performed following the manufacturer instructions. Briefly, 30‐μm coronal sections were dried at 65°C for 2 h, rehydrated, and then stained with Nissl Staining Solution (C0117, Beyotime) for 5 min at 37°C. The interval between successive slide groups was 90 μm. Five regions of each slice and three slices from each mouse were imaged and analyzed. An observer blinded to the experimental groups counted the total number of Nissl‐positive neurons in different regions of each slice and three slices from each mouse using a light microscope (BX51; Olympus).

### 2.9 TUNEL staining

The brain sections utilized for TUNEL staining were identical to those employed for Nissl staining. Briefly, the brain slices were treated with PBS containing 0.3% Triton and 10% donkey serum for 30 min. Following this, the slices were subjected to incubation with 50 μL of biotin labeling solution sourced from a commercially available fluorescent terminal deoxynucleotidyl transferase nick‐end labeling kit (TUNEL, Beyotime, C1090). All slices were mounted with mounting medium (Absin, abs9240). All sections were observed and photographed under a fluorescence microscope by an observer blinded to the experimental groups. ImageJ software was used to count cells, and quantification was carried out using three mice from each group.

### 2.10 RNA isolation and sequencing

After the mice were euthanized by neck removal, the hippocampus was immediately dissected and snap‐frozen in liquid nitrogen. Total RNA was extracted using TRIzol (Sangon), and the quality of RNA samples was assessed using a SMA4000 microspectrophotometer (Merinton). The extracted RNA samples were then sequenced on a NovaSeq6000 (Illumina) platform at Shanghai Sangon. The “edgeR” algorithm was employed to identify differentially expressed genes, and the “clusterProfiler” package in R language was utilized for GO analysis of differentially expressed genes. Both *p*‐values and *q*‐values were set at 0.05.

### 2.11 Morris water maze

The Morris water maze (MWM) was conducted 2 weeks following TBI to assess the learning and memory of the mice as previously described.^19^ The maze was a circular pool that was 50 cm high and 120 cm wide. It had an escape platform with a 6 cm diameter, located 1 cm below the water surface. The water was consistently heated and made white using titanium dioxide. Mice swam in the pool to find the platform, with multiple starting locations per trial. If they did not find the platform within 90 s, they were directed toward it. Mice were tested in four trials per day, for six consecutive days, with a short break between each. On the seventh day, a probe trial was conducted to measure memory. The trajectories of the mice were analyzed by the video‐tracking system (DigBehv, Jiliang Software Technology Company).

### 2.12 Statistical analysis

The statistical analysis of the data was performed using GraphPad Prism software (version 9.0). Each set of data was collected using at least three biological replicates, and the results are presented as the mean and standard error of the mean (SEM). Normality was assessed with the Shapiro–Wilk test. One‐way ANOVA was conducted to examine differences between multiple groups, and a post hoc test was applied to compare means among multiple groups. The Chi‐square test was used for nonparametric data. The significance level was set at *p* < 0.05.

## 3 RESULTS

### 3.1 Dynamic changes in glutamate carboxypeptidase expression after TBI

**FIGURE 1:**
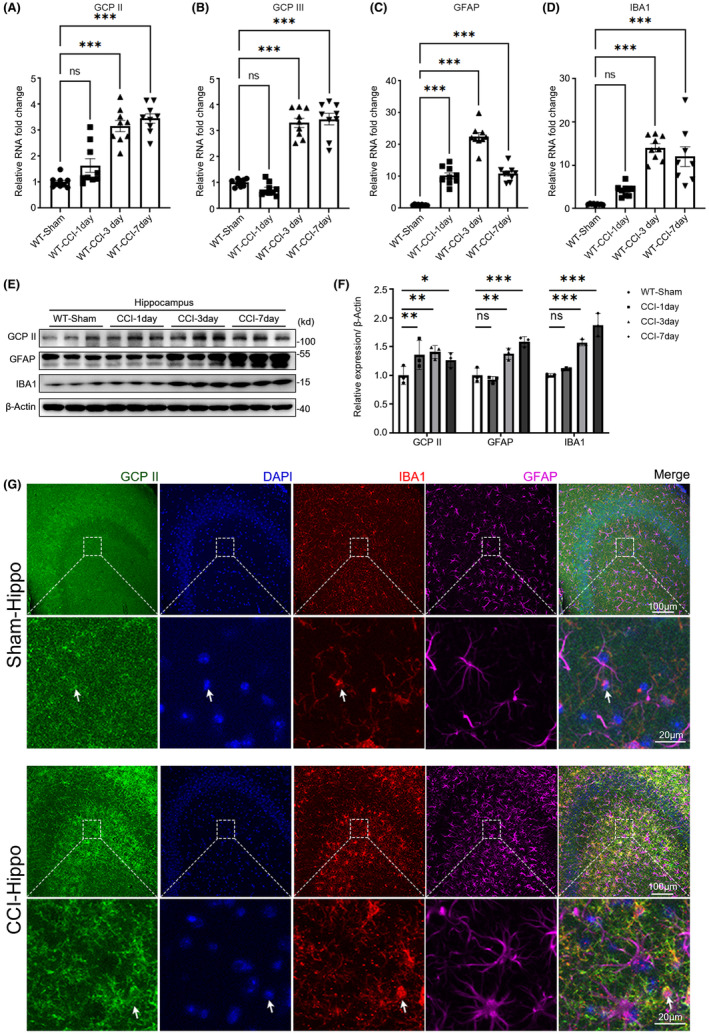
Dynamic changes in glutamate carboxypeptidase expression after TBI. (A–D) Quantitative reverse transcription‐polymerase chain reaction (RT–PCR) was used to assess the mRNA expression levels of GCPII (A), GCPIII (B), GFAP (C), and IBA1 (D) in the injured hippocampus at 1, 3, and 7 days after TBI and in the same brain area in the Sham operation group (*n* = 3 per group, one‐way ANOVA, *: versus WT‐Sham group). The mRNA expression of GCPII and GCPIII in the injured hippocampus gradually increased from day 3 after injury and remained elevated for at least 7 days after injury. (E, F) Representative immunoblots and quantification showing the protein expression levels of GCPII, GFAP, and IBA1 in the injured hippocampus at 1, 3, and 7 days after TBI and in the same brain area in the Sham operation group. The data are expressed as the fold change compared to the Sham operation group (*n* = 3 per group, one‐way ANOVA, *: versus WT‐Sham group). (G) Representative images of GCPII, IBA1, and GFAP co‐staining in the injured hippocampus after TBI and in the same brain areas in the Sham operation group showing that GCPII was abundantly expressed in the plasma membrane of microglia and substantially upregulated in activated microglia following TBI (scale bar = 100 μm). The magnified regions are indicated by the boxes (scale bar = 20 μm). White arrows indicate cells clearly expressing GCPII. The errors bars indicate the S.E. ***p < 0.001, **p < 0.01, and *p < 0.05.

RT‐qPCR and Western blotting demonstrated that the expression of GCPII, GCPIII, GFAP, and IBA1 in the injured hippocampus was significantly increased in the TBI group compared to the Sham group on the third and seventh days after injury (Figure 1A–D). Similar results were observed in the injured cortex (Figure S1A–D). As there is no specific monoclonal antibody against GCPIII,^20^ the protein levels of GCPII, GFAP, and IBA1 were further analyzed in the injured hippocampus and cortex at different time points after TBI (Figure 1E,F; Figure S1E,F). The expression of GCPII, GFAP, and IBA1 was significantly increased after 3 days of TBI and peaked around day 7. To compare the cellular localization of GCPII in the control and injury groups, GCPII/GFAP/IBA1 co‐staining was performed on the ipsilateral hippocampus. The results showed that GCPII was abundantly expressed in the plasma membrane of microglia in the Sham group, and its expression was substantially upregulated in activated microglia with phagocytic morphology at 7 days post‐TBI (Figure 1G). Similar findings were observed in the cortex (Figure S1G).

### 3.2 Generation of GCPII, GCPIII, and GCPII/III gene knockout mice by CRISPR/Cas9‐mediated genome engineering

**FIGURE 2:**
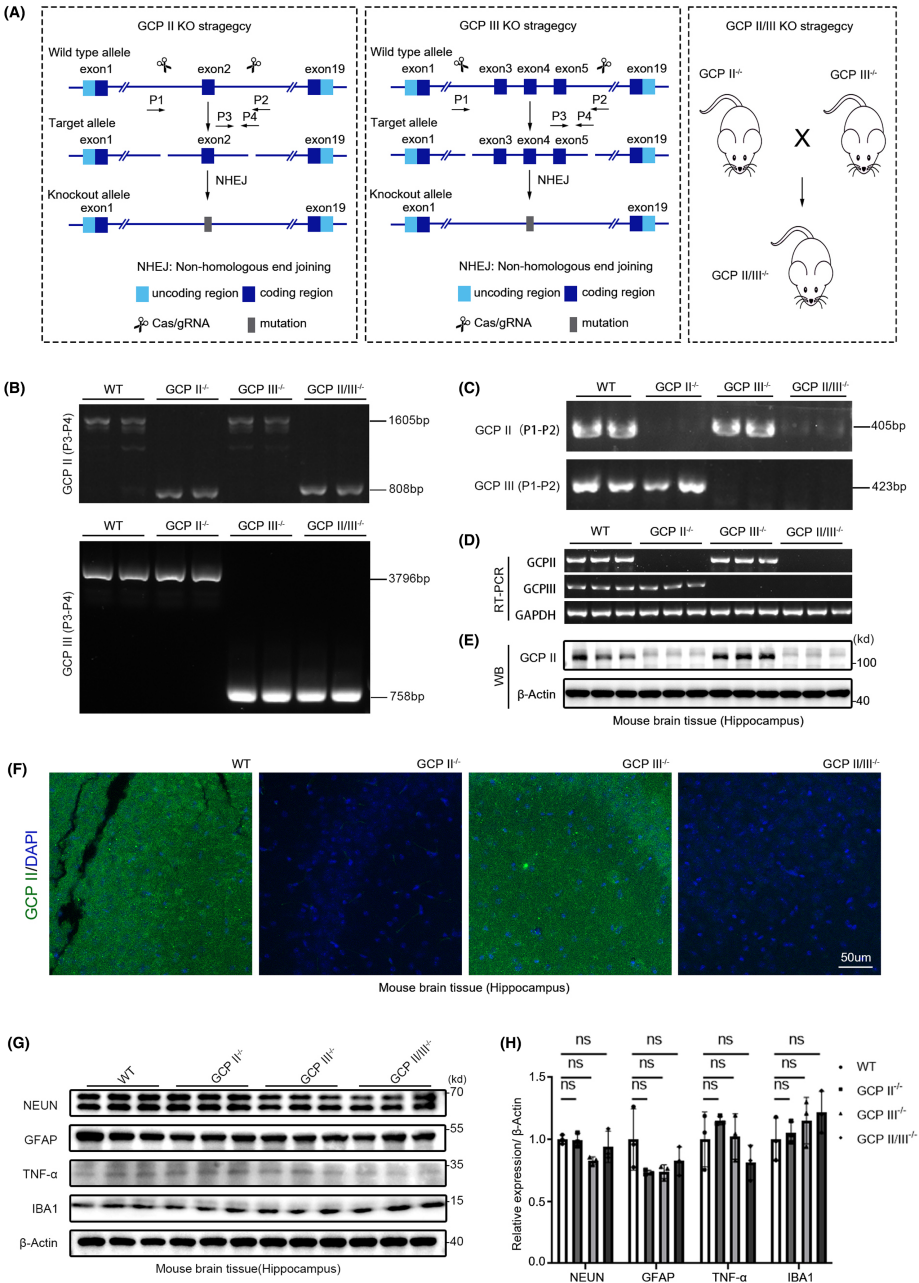
Generation of GCPII, GCPIII, and GCPII/III gene knockout mice by CRISPR/Cas9‐mediated genome engineering. (A) Schematic of the strategy used to generate GCPII, GCPIII, and GCPII/III knockout mice. (B, C) Genotyping of GCPII, GCPIII, and GCPII/III knockout mice by RT–PCR analysis of tail DNA samples. (B) The molecular weight of the PCR product of GCPII was 1605 bp in wild‐type mice and GCPIII^−/−^ mice and 808 bp in GCPII^−/−^ and GCPII/III^−/−^ mice (GCPII, P3 and P4 primers), while the molecular weight of the PCR product of GCPIII was 3796 bp in wild‐type and GCPII^−/−^ mice and 758 bp in GCPII^−/−^ and GCPII/III^−/−^ mice (GCPIII, P3 and P4 primers). (C) The molecular weight of the PCR product of GCPII was 405 bp in wild‐type and GCPIII^−/−^ mice (GCPII, P1 and P2 primers). No band was detected in GCPII^−/−^ and GCPII/III^−/−^ mice. The molecular weight of the PCR product of GCPIII was 423 bp in wild‐type mice and GCPII^−/−^ mice (GCPIII, P1 and P2 primers). No band was detected in GCPIII^−/−^ and GCPII/III^−/−^ mice. (D) RT–PCR analysis of GCPII and GCPIII expression in the hippocampus of wild‐type, GCPII^−/−^, GCPIII^−/−^, and GCPII/III^−/−^ mice. The molecular weight of the PCR product of GCPII was 405 bp in wild‐type and GCPIII^−/−^ mice (GCPII, P1 and P2 primers). No band was detected in GCPII^−/−^ and GCPII/III^−/−^ mice. The molecular weight of the PCR product of GCPIII was 423 bp in wild‐type mice and GCPII^−/−^ mice. No band was detected in GCPIII^−/−^ and GCPII/III^−/−^ mice (GCPIII, P1 and P2 primers). Gapdh was used as an endogenous reference gene. (E) Western blot analyses of GCPII expression in the hippocampus of wild‐type, GCPII^−/−^, GCPIII^−/−^, and GCPII/III^−/−^ mice. A band over 100 kDa was detected in the wild‐type and GCPIII^−/−^ mice, while no GCPII protein was detected in GCPII^−/−^ and GCPII/III^−/−^ mice. β‐Actin was used to control for loading. (F) Immunofluorescence, GCPII (green) in the hippocampus of wild‐type, GCPII^−/−^, GCPIII^−/−^, and GCPII/III^−/−^ mice. Scale bar = 50 μm. (G) The expression levels of NEUN, GFAP, TNF‐α, and IBA1 in hippocampus of wild‐type, GCPII^−/−^, GCPIII^−/−^, and GCPII/III^−/−^ mice were detected by immunoblotting, and β‐Actin was used to control for loading. The right panel (H) is the quantitative analysis of the protein bands (*n* = 3 per group, one‐way ANOVA, *p* > 0.05 among all groups). The errors bars indicate the S.E.

The CRISPR–Cas9 system has proven to be efficient in creating knockout mice for examining physiological functions and pathological mechanisms.^21^, ^22^, ^23^ Thus, we utilized this system to generate GCPII knockout mice (GCPII^−/−^), GCPIII knockout mice (GCPIII^−/−^), and GCPII/III^−/−^ mice to investigate the impact of their deletion in the brain after TBI. Guide RNAs were designed to target specific exons to create these mice (Figure 2A). Genotyping from the mice's tail tips was done to confirm the appropriate deletion of GCPII and GCPIII with PCR (Figure 2B,C). Additionally, RT‐PCR using primers targeting GCPII or GCPIII revealed truncated messenger RNA in the hippocampus and cortex of the mutant mice (Figure 2D; Figure S2A).

To confirm the GCPII protein's deletion, Western blot (WB) analysis was performed on the cortex and hippocampus of wild‐type, GCPII^−/−^, GCPIII^−/−^ and GCPII/III^−/−^ mice. The GCPII band was visible in the hippocampus and cortex of wild‐type and GCPIII^−/−^ mice but not in GCPII^−/−^ and GCPII/III^−/−^ mice (Figure 2E; Figure S2B). Immunofluorescence also confirmed the loss of GCPII protein (Figure 2F; Figure S2C). Besides, no discernible difference was observed in protein expression of NEUN, GFAP, TNF‐α, and IBA1 between wild‐type and gene knockout mice hippocampus or cortex (Figure 2G,H; Figure S2D,E), demonstrating the successful construction of GCPII, GCPIII, and GCPII/III knockout mice.

### 3.3 GCPII and GCPII/III knockout attenuated glutamate excitotoxicity

**FIGURE 3:**
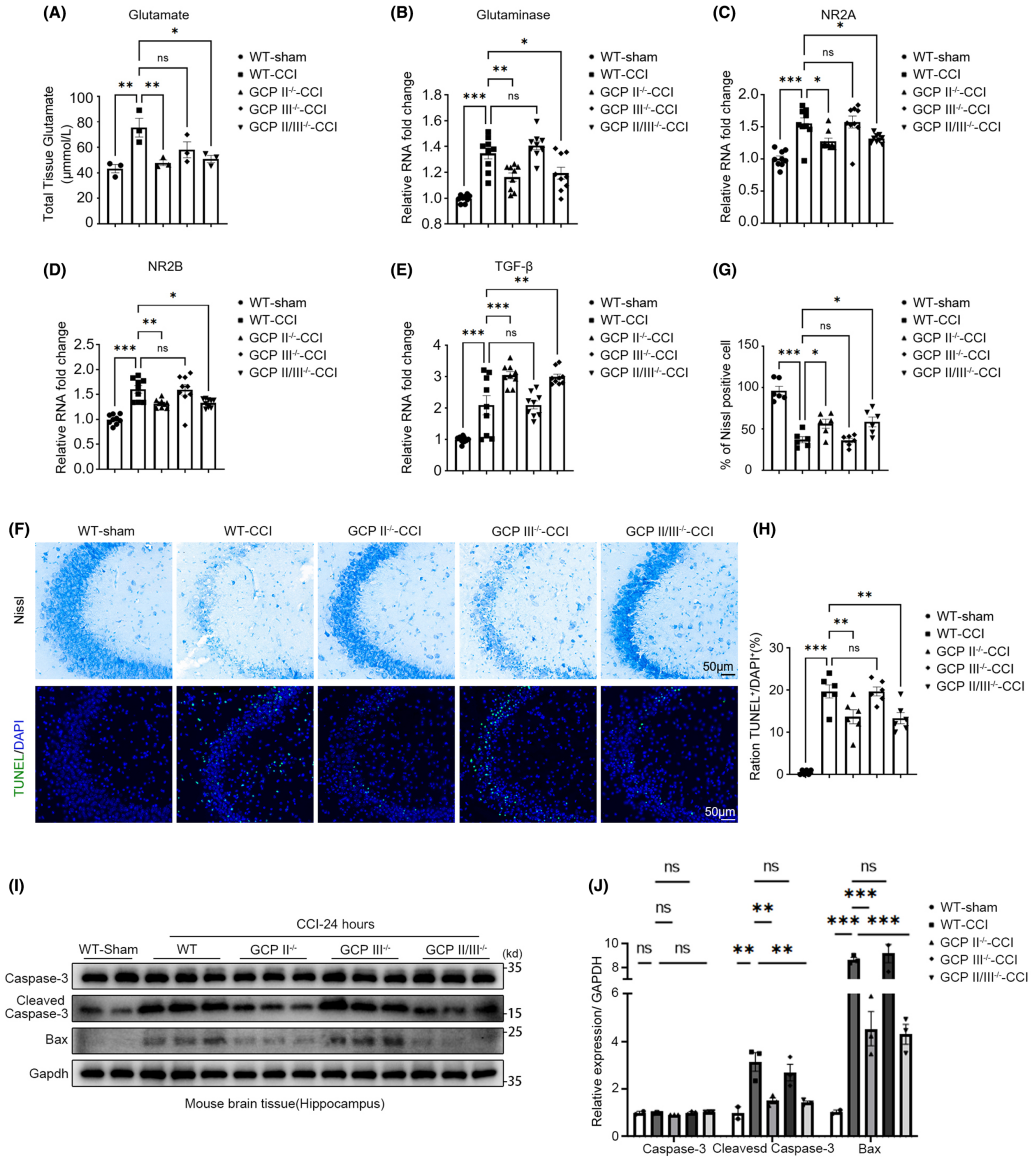
GCPII and GCPII/III knockout attenuated glutamate excitotoxicity. (A) The glutamate concentration (μmol/L) in the hippocampus was significantly increased in the injury group compared with the Sham group at 24 h post‐TBI, while the concentration of glutamate in the injured hippocampus was significantly decreased in GCPII^−/−^ and GCPII/III^−/−^ mice, but not GCPIII^−/−^ mice, compared with the WT‐CCI group (*n* = 6 per group, one‐way ANOVA, *: versus WT‐CCI group). (B–E) The mRNA expression of glutaminase, NR2A, NR2B, and TGF‐β were measured by RT‐qPCR. The mRNA levels of glutaminase and glutamate receptors (NR2A and NR2B) in the hippocampus were significantly increased in the injury group compared with the Sham group at 24 h post‐TBI and were decreased in GCPII^−/−^ and GCPII/III^−/−^ mice, but not GCPIII^−/−^ mice, compared with the WT‐CCI group. (E) Compared with the injury group, GCPII^−/−^ and GCPII/III^−/−^ mice had significantly increased TGFβ‐1 mRNA expression (*n* = 9 per group, one‐way ANOVA, *: versus WT‐CCI group). (F–H) 24 h post‐TBI, Nissl staining (G) and TUNEL (H) staining were used to analyze the number of neurons and apoptotic cells in hippocampal CA3 region of WT‐Sham, WT‐CCI, GCPII^−/−^‐CCI, GCPIII^−/−^‐CCI, and GCPII/III^−/−^‐CCI mice (scale bar = 50 μm, *n* = 6 per group, one‐way ANOVA, *: versus WT‐CCI group). (I) 24 h post‐TBI, the expression levels of Caspase3, cleaved Caspase3, and Bax in the injured hippocampus were detected by Western blot. Gapdh was used as an internal control. The right panel (J) is a quantitative analysis of the Western blot bands (*n* = 3 per group, one‐way ANOVA, *: versus WT‐CCI group). The error bars indicate the S.E. ***p < 0.001, **p < 0.01, and *p < 0.05.

On the first day following TBI, there was a notable rise in glutamate concentration in the hippocampus of the TBI group compared to the Sham group, while GCPII^−/−^ and GCPII/III^−/−^ mice displayed significantly lower levels of glutamate in comparison to the WT‐CCI group (Figure 3A). Furthermore, glutaminase and NMDA receptor subunits NR2A and NR2B mRNA expression were measured in the hippocampus on the first day after TBI. It was found that their expression was significantly increased in the TBI group compared to the Sham group and significantly decreased in the GCPII^−/−^ and GCPII/III^−/−^ group compared to the WT‐CCI group, but no significant differences were observed between GCPIII^−/−^ group and WT‐CCI group (Figure 3B–D).

Activation of mGluR3 regulates TGFβ1 signaling, which in turn modulates inflammation and cell death in TBI.^24^, ^25^ TGFβ1 mRNA in the injured hippocampus was significantly upregulated in the TBI group compared to the Sham group and markedly increased in GCPII^−/−^ and GCPII/III^−/−^ mice compared to the WT‐CCI group on the first day following TBI (Figure 3E).

Nissl and TUNEL staining were used to evaluate neuronal damage and cell apoptosis. It was found that GCPII deletion and GCPII/III deletion, but not GCPIII deletion, alleviated neuronal damage (Figure 3F,G) and decreased the number of apoptotic cells on the first day after TBI (Figure 3F,H).

Besides, WB revealed significantly elevated levels of Bax and cleaved Caspase‐3 in the TBI group compared to the WT‐Sham group, and these changes were reversed in the GCPII and GCII/III knockout mice (Figure 3I,J).

### 3.4 GCPII and GCPII/III knockout led to long‐term neuroprotective effects

The transition from the acute to subacute phase after TBI is a crucial pathophysiological process that involves changes in various nerve cells, chemokines, and signal transduction.^26^, ^27^ Thus, we assessed the cell state in the hippocampus and cortex around the injured area in each genotype of mice during the subacute phase after TBI (7th day post TBI).

**FIGURE 4:**
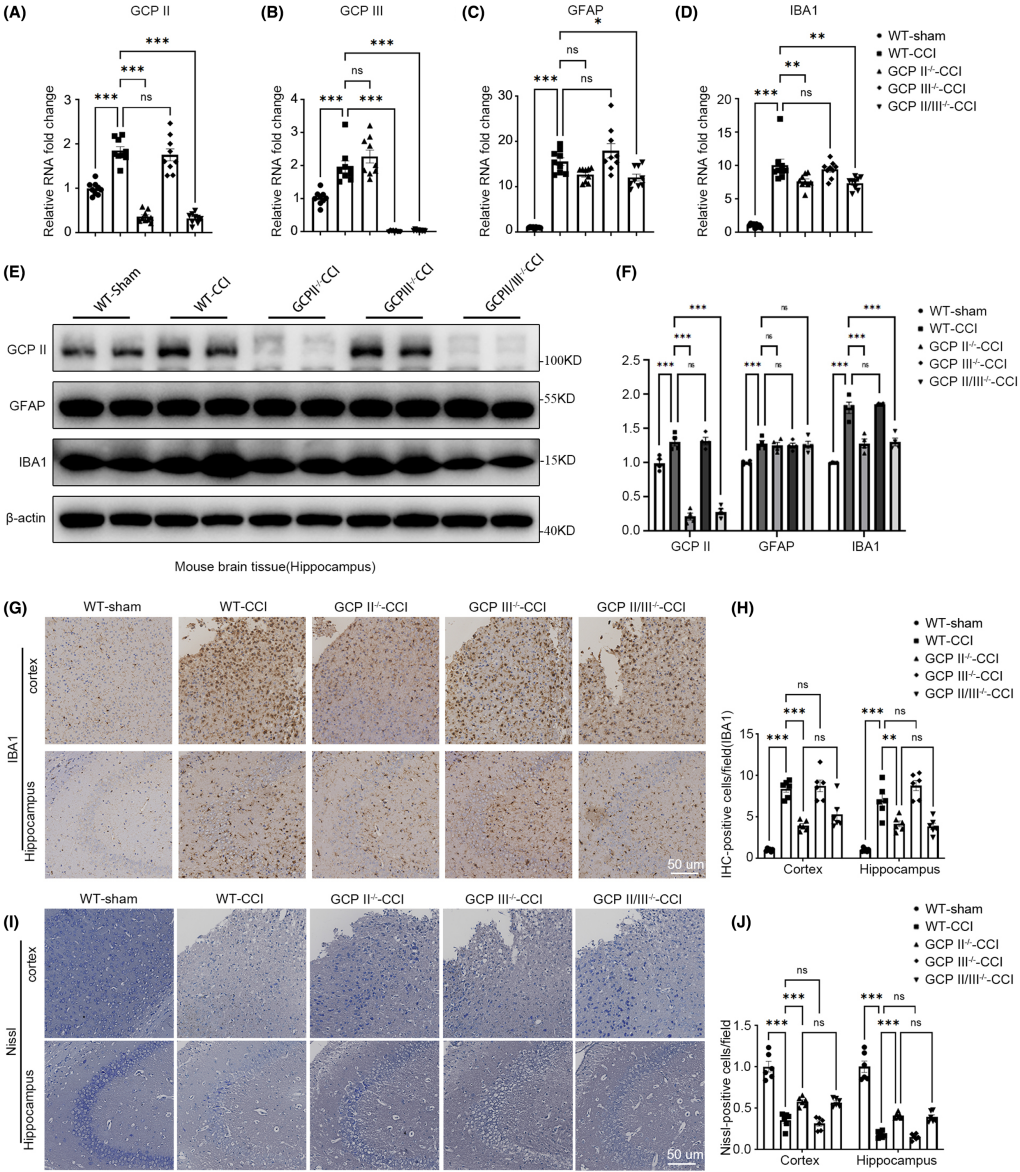
GCPII and GCPII/III knockout led to long‐term neuroprotective effects. To explore the microscopic changes in the brain of mice with different genotypes in the subacute stage of TBI (7 days after TBI). (A–D) RT‐qPCR was used to detect the mRNA expression of GCPII, GCPIII, GFAP, and IBA1 in the hippocampal CA3 region of the WT‐Sham, WT‐CCI, GCPII^−/−^‐CCI, GCPIII^−/−^‐CCI, and GCPII/III^−/−^‐CCI mice (*n* = 9 per group, one‐way ANOVA, *: versus WT‐CCI group). (E) The protein expression levels of GCPII, GFAP, and IBA1 in the injured hippocampus of each group were detected by Western blot. The right panel (F) is a quantitative analysis of the protein bands, suggesting decreased IBA protein expression in GCPII^−/−^‐CCI and GCPII/III^−/−^‐CCI mice (*n* = 3 per group, one‐way ANOVA, *: versus WT‐CCI group). Immunohistochemical staining of IBA1 (G) and Nissl staining (I) were performed on the cortex around the injury site and the CA3 region of the hippocampus of each group mice, respectively (scale bar = 50 μm, *n* = 6 per group, one‐way ANOVA, *: versus WT‐CCI group), suggesting that GCPII^−/−^‐CCI and GCPII/III^−/−^‐CCI mice had reduced microglial activation (H) in the subacute phase of TBI, as did the number of dead neurons (J). The error bars indicate the S.E. ***p < 0.001, **p < 0.01, and *p < 0.05.

In comparison to the Sham group, GCPII, GCPIII, GFAP, and IBA1 mRNA were significantly increased in hippocampus (Figure 4A–D). Based on the analysis of PCR and WB, IBA1 in GCPII^−/−^ and GCPII/III^−/−^ hippocampus, but not in GCPIII^−/−^, was proved to express significantly lower than the WT‐CCI group (Figure 4E,F). HE staining confirmed that GCPII^−/−^ and GCPII/III^−/−^ mice exhibited reduced cortical and hippocampal microglial activation (Figure 4G,H), while astrocyte activation was not statistically different among all injury groups of mice (Figure S3A,B).

Furthermore, Nissl and TUNEL staining were utilized to evaluate the neuron status and cell apoptosis in the cortex and hippocampus. In comparison to the WT‐CCI group, GCPII^−/−^ and GCPII/III^−/−^ mice showed a statistically significant recovery in the number of neurons (Figure 4I,J) and reduction in the number of apoptotic cells (Figure S3C,D). There were no statistical differences between WT‐CCI and GCPIII^−/−^ or between GCPII^−/−^ and GCPII/III^−/−^ mice.

### 3.5 Glutamate carboxypeptidase knockout affected global gene expression in the hippocampus after TBI

The response of GCPII knockout mice to brain injury in the subacute phase of TBI has not been well studied, nor has the response of GCPIII knockout mice been reported. Therefore, whole‐transcriptome sequencing of the injured hippocampus in each group on day 7 after TBI was performed to further elucidate the differences in gene expression and signaling pathway changes among WT‐Sham, WT‐CCI, GCPII^−/−^, GCPIII^−/−^, and GCPII/III^−/−^ group.

**FIGURE 5:**
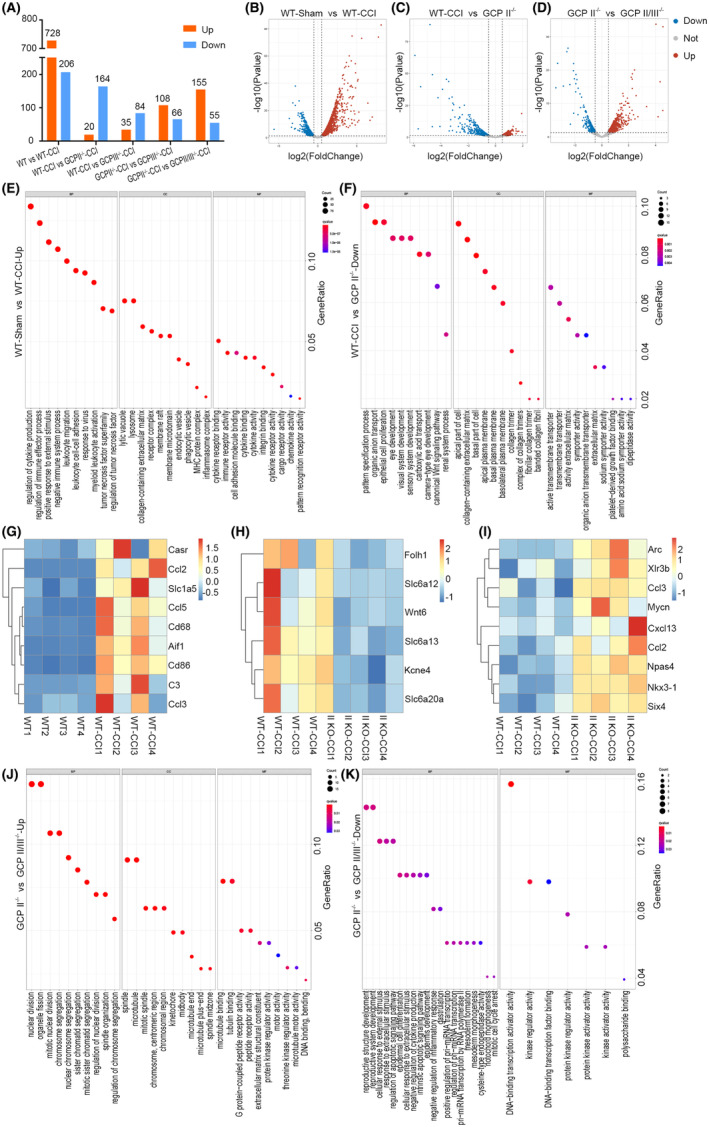
Glutamate carboxypeptidase knockout affected global gene expression in the hippocampus after TBI. The hippocampus at the injured side were collected for whole‐transcriptome sequencing on the seventh day post‐TBI (*n* = 4 per group, p‐value and q‐value were set at 0.05). (A) Differentially expressed genes among groups were screened based on “edgeR” algorithm. A total of 728 genes were significantly upregulated in the WT‐CCI group compared with the Sham group, while 164 genes were significantly downregulated in the GCPII^−/−^‐CCI group compared with the WT‐CCI group. Volcano plot (B) showed that a large number of genes were significantly upregulated in the WT‐CCI group compared with the Sham group, while GCPII knockout (C) could significantly reverse the upregulation trend of many genes. In addition, there were many differentially expressed genes between GCPII^−/−^‐CCI and GCPII/III^−/−^‐CCI groups (D). GO analysis results were ranked from high to low by GeneRadio, and the top 10 enrichment results were selected. (E) Gene Ontology (GO) analysis of the upregulated genes between the WT‐CCI group and the Sham group indicated that immune‐related functions were significantly active. Part of these upregulated genes were selected to draw the heatmap (G) after the standardization of gene expression and k‐means clustering, and it was found that immune‐related chemokines, microglia, and astrocyte‐related genes were significantly upregulated. (F) GO analysis of downregulated genes between the GCPII^−/−^‐CCI and the WT‐CCI group indicated the functions related to transmembrane transport and sodium ion transport. Some key genes were screened out to draw the heatmap (H, I), it was found that the expression of some solute carrier family 6 (Slc6) members was downregulated, and some functional genes such as Wnt6 and Kcne4 were also downregulated. The upregulated genes included not only some chemokines (Ccl2 and Ccl3) but also synaptic repair‐related gene Npas4. (J, K) GO analysis was performed on the differentially expressed genes between GCPII/III^−/−^‐CCI group and GCPII^−/−^‐CCI group. The result suggested that there may be differences in cell cycle, apoptosis regulation, and DNA damage response between these two genotypes mice.

Seven hundred and twenty‐eight genes were significantly upregulated in the WT‐CCI group compared with the Sham group (Figure 5A,B), and one hundred and sixty–four genes were significantly downregulated in the GCPII^−/−^ group compared with the WT‐CCI group (Figure 5A,C). There were different numbers of differentially expressed genes between the other groups (Figure 5A,D, Figure S4A,B).

Compared with the Sham group, a large number of immune, inflammatory, and chemoattraction‐related genes were increased in the WT‐CCI group, such as Ccl2, Ccl3, Ccl5, Cd68, Aif1, Cd86, and C3 (Figure 5E,G). GO analysis of downregulated genes between the GCPII^−/−^ and WT‐CCI group indicated the functions related to transmembrane transport and sodium ion transport (Figure 5F). Solute carrier family 6 (Slc6a12, Slc6a13, and Slc6a20a), Wnt6, Kcne4, etc., were downregulated (Figure 5H). The upregulated genes between the GCPII^−/−^ and WT‐CCI group included not only some chemokines (Ccl2 and Ccl3) but also the synaptic repair‐related gene Npas4 (Figure 5I). It was suggested that there may be differences in cell cycle, apoptosis regulation, and DNA damage response between GCPII^−/−^ and GCPII/III^−/−^ group (Figure 5J,K).

Furthermore, GO analysis between the GCPIII^−/−^ and GCPII^−/−^ group indicated the functions related to angiogenesis regulation, inflammatory response, p38MAPK cascade regulation, and forebrain development (Figure S4C,D). Downregulated genes between the GCPIII^−/−^ and WT‐CCI group indicated the functions related to epithelial development, angiogenesis, and DNA‐binding transcription activator activity (Figure S4E).

### 3.6 Effects of glutamate carboxypeptidase knockout on the learning and memory of mice

We conducted the fixed platform version of the MWM to investigate the effect of GCPII and GCPIII knockout on mice cognition after injury.

**FIGURE 6:**
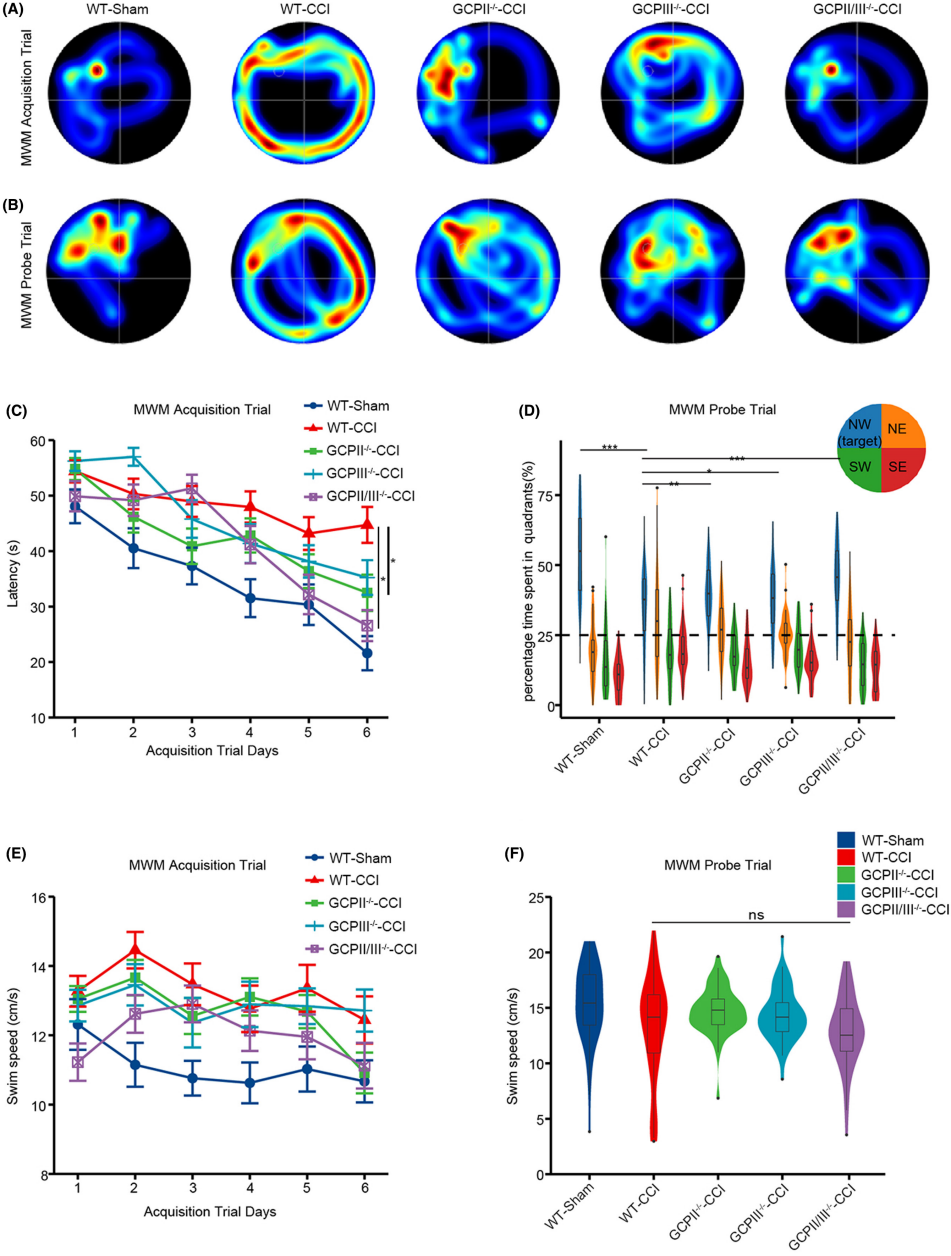
Effects of glutamate carboxypeptidase knockout on the learning and memory of mice. (A) Representative heatmap images of the WT‐Sham, WT‐CCI, GCPII^−/−^‐CCI, GCPIII^−/−^‐CCI, and GCPII/III^−/−^‐CCI groups in the acquisition trial. The longer the mice spent in each quadrant, the redder the image displayed. Conversely, a cooler color indicates a shorter duration. (B) Representative heatmap images of the WT‐Sham, WT‐CCI, GCPII^−/−^‐CCI, GCPIII^−/−^‐CCI, and GCPII/III^−/−^‐CCI groups in the probe trial. (C) The latency to find the escape platform of the different groups was measured. After TBI, a significant memory deficit was observed in the WT‐CCI group compared with the Sham group. Notably, compared with the WT‐CCI group, the GCPII^−/−^‐CCI group, and GCPII/III^−/−^‐CCI group showed a significant improvement with reduced latency (*n* = 11–14 per group, two‐way ANOVA, *: versus WT‐CCI group). (D) The time spent in four quadrants by different groups mice was measured. Mice in the WT‐Sham, GCPII^−/−^‐CCI, and GCPII/III^−/−^‐CCI groups showed spatial memory retention, as evidenced by their significantly higher percentage of time spent in the target quadrant (NW) than in all other quadrants. However, mice in the WT‐CCI and GCPIII^−/−^‐CCI groups did not show a preference for the target quadrant compared with the other quadrants (*n* = 11–14 per group, two‐way ANOVA, *: versus WT‐CCI group). The swimming speed of different group mice on all acquisition days (E) and in the probe trial (F) was measured. The error bars indicate the S.E. ***p < 0.001, **p < 0.01, and *p < 0.05.

During the acquisition trials, mice subjected to TBI required significantly more time to find the platform than Sham group. The latency to find the platform was significantly reduced in GCPII^−/−^ and GCPII/III^−/−^ group compared to WT‐CCI group. (Figure 6A,C).

In the probe test, percentage of time spent in the target quadrant demonstrated a significant difference between TBI and Sham group, while GCPII^−/−^ and GCPII/III^−/−^ mice spent more time in the target quadrant compared to WT‐CCI mice (*p* < 0.05, Figure 6B,D). On the other hand, it was observed that GCPIII^−/−^ mice exhibited spatial memory impairment, as evidenced by their absence of preference toward the target quadrant. There was no evidence of impaired motor function in any group, as swimming speed was not altered on any day of the acquisition phase or in the probe trial (Figure 6E,F).

## 4 DISCUSSION

Previous studies have reported that GCPII mRNA was primarily expressed in astrocytes,^28^ and GCPII protein was found in the neuropil,^29^ astrocytes,^30^, ^31^, ^32^, ^33^ and microglia.^30^, ^31^, ^34^ The current study confirmed that GCPII is constitutively expressed at low levels in microglia in the brains of adult mice, but its expression is significantly upregulated following brain injury (Figure 1). Using the maternal inflammation‐induced cerebral palsy rabbit model, Zhang et al. demonstrated that GCPII is localized in astrocytes and/or microglia following injury.^30^ The same research group provided evidence that GCPII is mainly localized in microglia in a superoxide dismutase (SOD) transgenic mouse model of experimental neonatal hypoxia‐ischemia (HI).^34^ The inconsistency in the data reported by this study and previous studies is likely due to differences in detection methods ranging from ELISA, WB, and immunohistochemistry to in vivo imaging.^35^ Besides, different antibodies were used and most antibodies were poorly characterized, which may have led to inappropriate use and false‐positive/‐negative results. The monoclonal antibody against GCPII used in this study was characterized by WB and IF to confirm its specificity in the brain. The data from this study demonstrated that GCPIII is expressed in the cortex and hippocampus, which is consistent with previous reports.^20^ However, the lack of a specific antibody against GCPIII limits rigorous investigation.

We utilized GCPII^−/−^, GCPIII^−/−^, and GCPII/III^−/−^ mice, which were generated using CRISPR/Cas9‐mediated genome engineering, to investigate the specific role of glutamate carboxypeptidase in the pathophysiology of TBI. The animals were subjected to experimental CCI injury, which is the most commonly used approach to model traumatic head injury. While our study focused on the hippocampus, it is important to note that secondary injuries in other brain regions, such as the cerebral cortex and corpus callosum, can also significantly impact the outcome of TBI.^36^ A significant body of evidence suggests that inhibition of GCPII by inhibitors provides substantial neuroprotection in various neuropathological disorders, including cerebral ischemia, spinal cord and TBI, inflammatory and neuropathic pain, motoneuron disease, peripheral neuropathy, epilepsy, and drug abuse.^34^, ^35^, ^37^, ^38^ Mice with deletion of the Folh1 (which encodes the GCPII enzyme) were generated and found to exhibit normal neurodevelopment.^9^, ^12^, ^13^ These gene knockout mice have been reported to be less susceptible to oxidative stress, neural damage, and impairment of cognitive functions after experimental TBI.^9^

In this study, we demonstrated that GCPII knockdown provided long‐term neuroprotection after TBI. Compared to the WT‐CCI group, GCPII^−/−^ and GCPII/III^−/−^ mice showed significantly decreased glutamate concentration and NMDA receptor expression and increased TGFβ1 signaling in the hippocampus at the acute stage of TBI (1 day after injury), along with significantly improved neuronal number and state, and a reduction in local apoptotic signals and apoptotic cells (Figure 3). These findings suggest that the excitotoxicity of nerve cells mediated by the change of glutamate concentration after TBI is effectively alleviated by GCPII knockdown.

In the sub‐acute phase of TBI (7 days after injury), GCPII^−/−^ and GCPII/III^−/−^ mice showed a significant reduction in microglia activation compared to the WT‐CCI group, in addition to the improvement in neuronal number and state (Figure 4). Microglia activation is a critical part of the secondary pathological process of TBI, mediating a series of inflammatory responses and regulating astrocyte activation.^39^ These results suggest that GCPII knockout still has a significant neuroprotective effect in the sub‐acute phase after TBI.

Several factors may contribute to this long‐term neuroprotective effect. First, the partial blockade of glutamate excitotoxicity in the acute phase of TBI leads to less nerve damage signal transduction and less secondary pathophysiological processes.^40^ Second, GCPII knockout may lead to other signal changes that improve neuronal state during the pathological process after TBI.

To clarify our second hypothesis, we conducted whole‐transcriptome sequencing of hippocampal tissue on the injured side of each group of mice on day 7 after TBI (Figure 5). After comparing and analyzing the sequencing results between groups, we observed that even in the subacute phase of TBI, the WT‐CCI group had a large number of immune and inflammation‐related chemokines, cytokines, and other genes that were active, including M1 microglia (CD86) and M2 microglia (Aif1), as well as A1 astrocytes (C3).

Compared to the WT‐CCI group, GCPII^−/−^ mice showed significant downregulation of Kcne4, suggesting that GCPII knockout may continuously affect the expression of ion channel‐related genes. Notably, Npas4 was significantly upregulated in GCPII^−/−^ mice. Npas4 is a key transcription factor found in brain neurons that helps regulate the balance between excitatory and inhibitory neural circuits. It is crucial for contextual memory formation and contributes to the flexibility of neurons. Npas4 activates the transcription of BDNF in excitatory neurons and regulates the number of GABA‐releasing synapses, resulting in an increase in inhibitory synapses on excitatory neurons.^41^, ^42^ These findings suggest that GCPII knockout may contribute to synaptic repair in hippocampal neurons.

It was confirmed that GCPIII deletion did not have a similar neuroprotective effect as GCPII deletion. This could be attributed to the lower efficiency of GCPIII in hydrolyzing NAAG.^20^ Notably, the lack of statistical differences observed between the GCPII knockout and GCPII/III knockout groups implies that the elimination of GCPII does not lead to compensatory functional changes in the NAAG hydrolysis capacity of GCPIII. Therefore, it appears that GCPII and GCPIII have distinct and non‐overlapping roles in the metabolism of NAAG. This finding has significant implications for understanding the physiological processes that involve these enzymes and for developing targeted therapeutic interventions for disorders that affect NAAG metabolism.

As studies on GCPIII's function are scarce, we examined changes in gene expression patterns in the subacute phase after TBI in GCPIII^−/−^ mice. Our findings revealed that GCPIII^−/−^ mice had enriched epithelial development, angiogenesis, and DNA‐binding transcription activator activity compared to both WT‐CCI and GCPII^−/−^ mice. These results suggest that GCPIII may be related to these functions, and further experimental verification is needed.

At day 14 post‐TBI, behavioral studies using the MWM confirmed the conclusion drawn from molecular experiments. Specifically, the results confirmed that GCPII deletion, but not GCPIII, is the primary determinant of neuroprotection after TBI.

In conclusion, our study revealed a significant upregulation of glutamate carboxypeptidase expression following TBI and confirmed increased GCPII expression in microglia within the brain. We also demonstrated that deletion of GCPII, but not GCPIII, had a positive impact on reducing glutamate production, excitability toxicity, and neuronal injury during the acute phase of TBI. Moreover, the protective effect of GCPII deletion on hippocampal neurons in the subacute phase of TBI may be achieved by regulating Npas4. Collectively, these findings confirm that GCPII knockout provides long‐term benefits to mice with TBI, ultimately leading to improved cognitive function.

## CONFLICT OF INTEREST STATEMENT

The authors declare no competing interests.

## Supporting information


Figure S1.

Figure S2.

Figure S3.

Figure S4.

## Data Availability

The publicly available datasets used in this study can be obtained from their respective online. All datasets used in the present study are available from the corresponding author on reasonable request.
