# Supplementary figures and images for: Deletion of glutamate carboxypeptidase II (GCPII), but not GCPIII, provided long‐term benefits in mice with traumatic brain injury
Source: CNS Neurosci Ther. 2023 Jun 22;29(12):3786–801. doi: 10.1111/cns.14299 (PMC10651966; doi:10.1111/cns.14299)

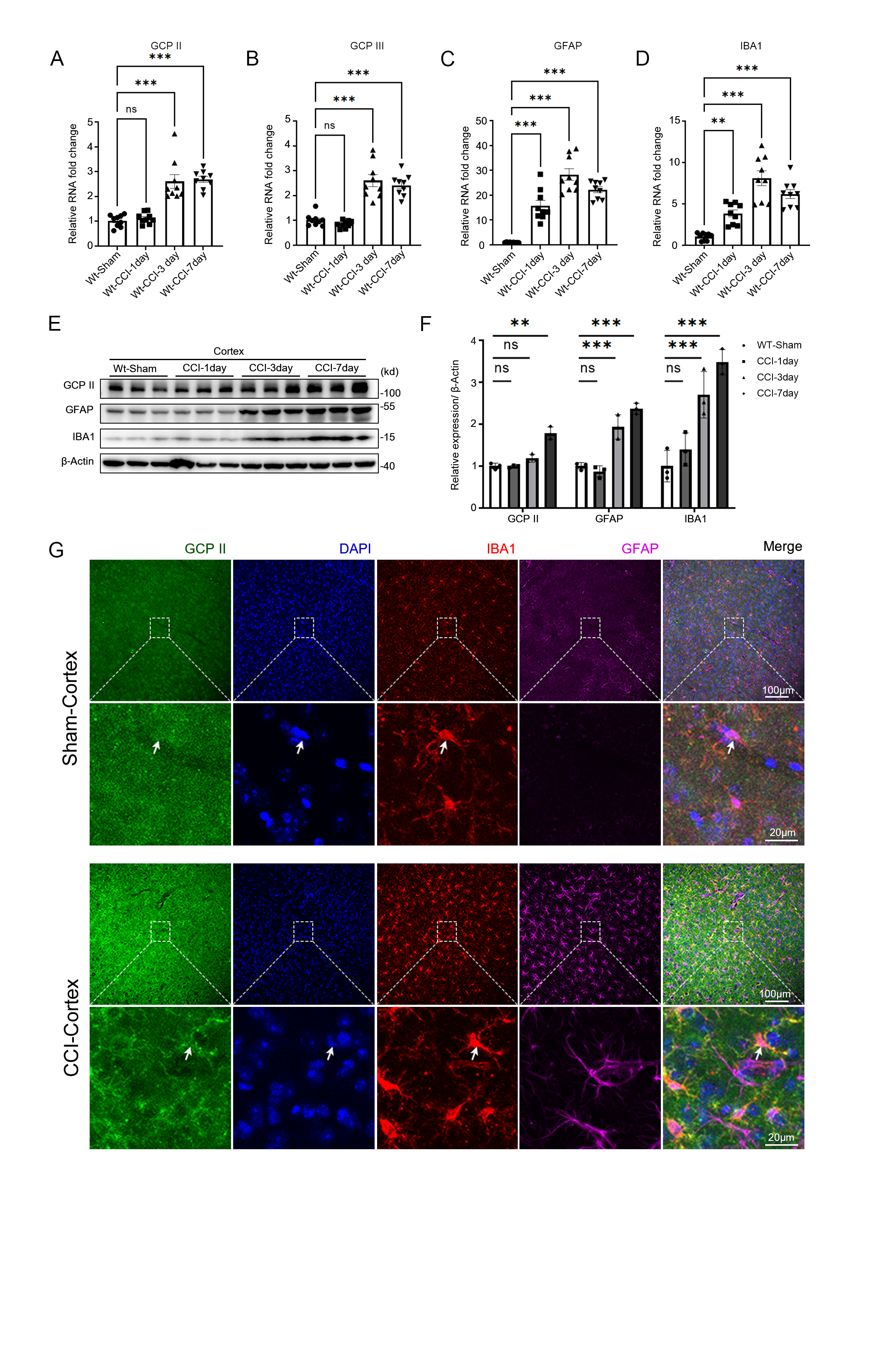

Supplement: Supplementary file 1 — Figure S1. [file CNS-29-3786-s002.jpg]

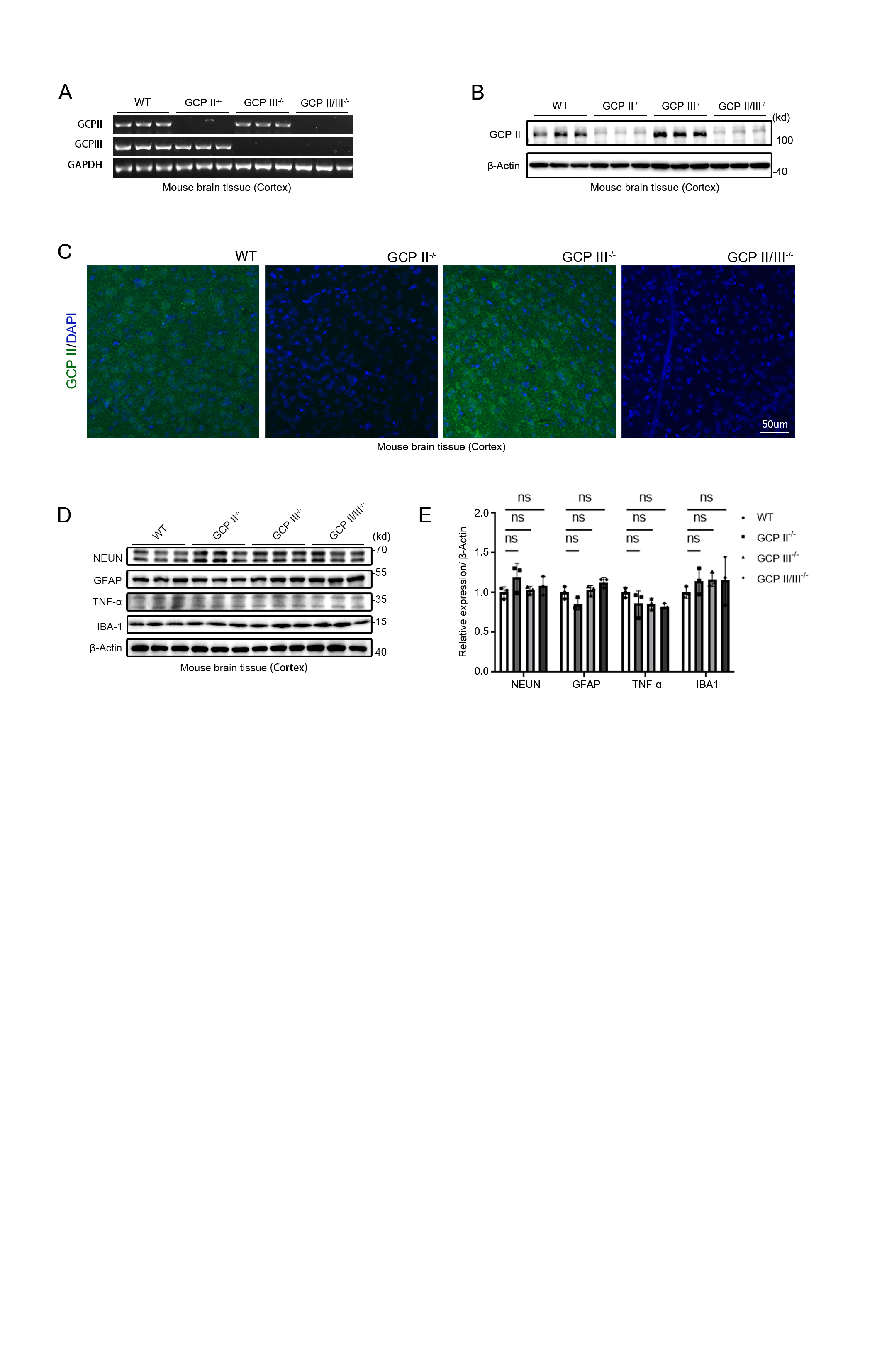

Supplement: Supplementary file 2 — Figure S2. [file CNS-29-3786-s003.jpg]

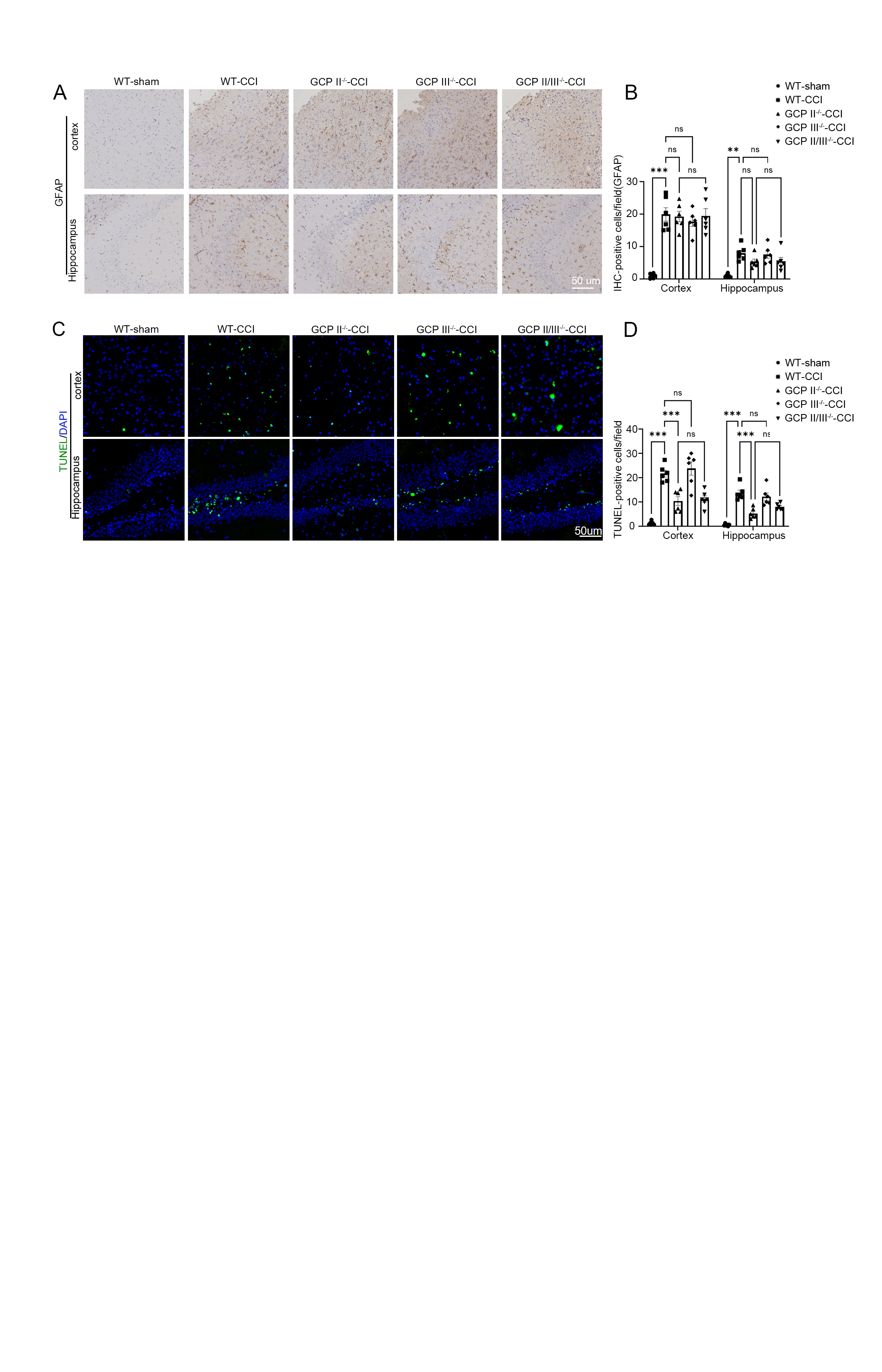

Supplement: Supplementary file 3 — Figure S3. [file CNS-29-3786-s004.jpg]

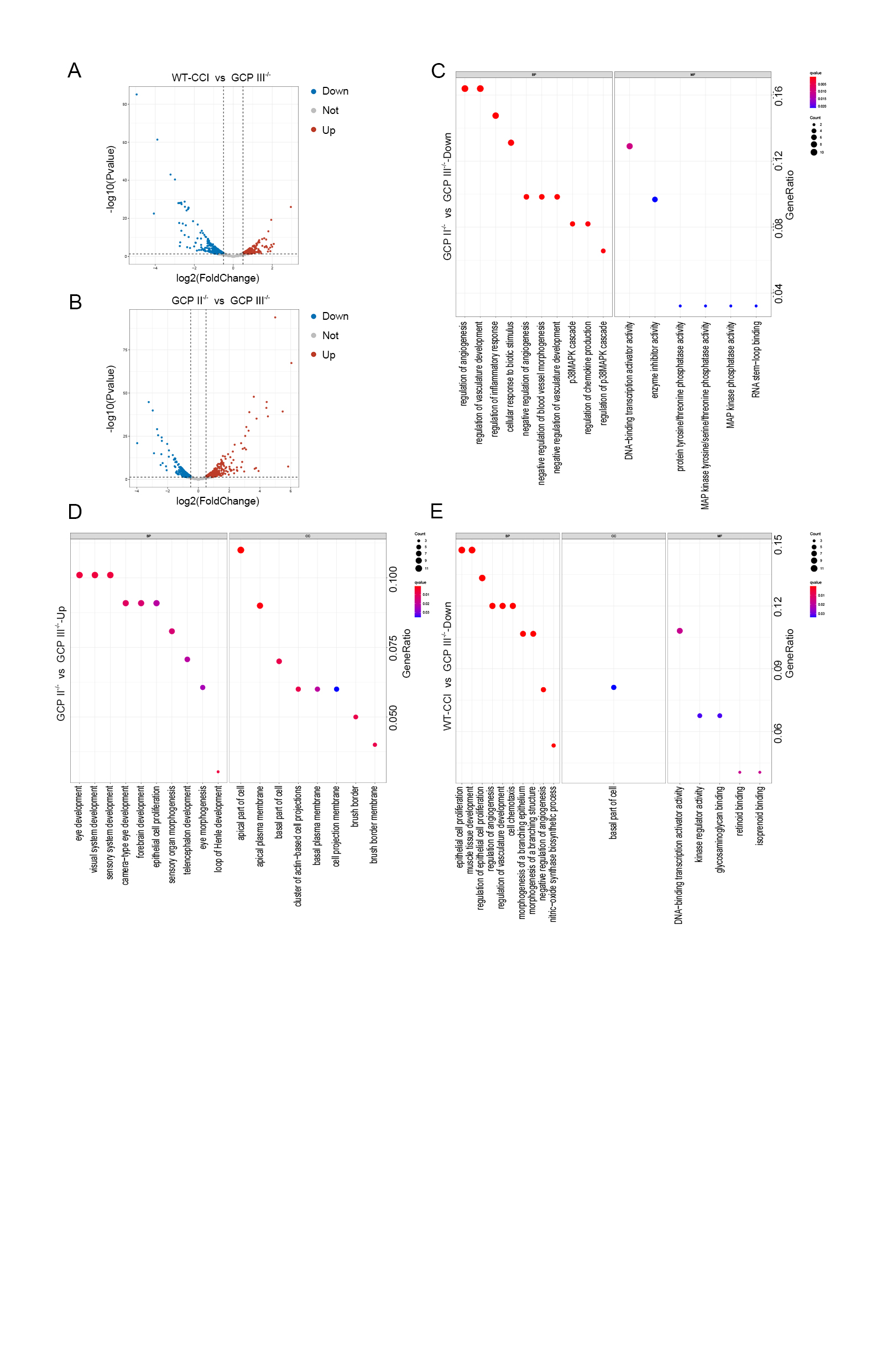

Supplement: Supplementary file 4 — Figure S4. [file CNS-29-3786-s001.jpg]
